# Experiences and Perceptions of Patients and Providers Participating in Remote Titration of Heart Failure Medication Facilitated by Telemonitoring: Qualitative Study

**DOI:** 10.2196/28259

**Published:** 2021-11-25

**Authors:** Veronica Artanian, Patrick Ware, Valeria E Rac, Heather J Ross, Emily Seto

**Affiliations:** 1 Institute of Health Policy, Management and Evaluation Dalla Lana School of Public Health University of Toronto Toronto, ON Canada; 2 Centre for Global eHealth Innovation Techna Institute University Health Network Toronto, ON Canada; 3 Program for Health System and Technology Evaluation Toronto General Hospital Research Institute University Health Network Toronto, ON Canada; 4 Ted Rogers Centre for Heart Research Peter Munk Cardiac Centre University Health Network Toronto, ON Canada; 5 Toronto General Hospital Research Institute University Health Network Toronto, ON Canada; 6 Department of Medicine University of Toronto Toronto, ON Canada

**Keywords:** telemonitoring, remote, titration, monitoring, mHealth, heart failure, qualitative, mobile phone

## Abstract

**Background:**

Guideline-directed medical therapy (GDMT), optimized to target doses, improves health outcomes in patients with heart failure. However, GDMT remains underused, with <25% of patients receiving target doses in clinical practice. A randomized controlled trial was conducted at the Peter Munk Cardiac Centre in Toronto to compare a remote GDMT titration intervention with standard in-office titration. This randomized controlled trial found that remote titration increased the proportion of patients who achieved optimal GDMT doses, decreased the time to dose optimization, and reduced the number of essential clinic visits. This paper presents findings from the qualitative component of the mixed methods study, which evaluated the implementation of the remote titration intervention.

**Objective:**

The objective of the qualitative component is to assess the perceptions and experiences of clinicians and patients with heart failure who participated in the remote titration intervention to identify factors that affected the implementation of the intervention.

**Methods:**

We conducted semistructured interviews with clinicians (n=5) and patients (n=11) who participated in the remote titration intervention. Questions probed the experiences of the participants to identify factors that can serve as barriers and facilitators to its implementation. Conventional content analysis was first used to analyze the interviews and gain direct information based on the participants’ unique perspectives. Subsequently, the generated themes were delineated and mapped following a multilevel framework.

**Results:**

Patients and clinicians indicated that the intervention was easy to use, integrated well into their routines, and removed practical barriers to titration. Key implementation facilitators from the patients’ perspective included the reduction in clinic visits and daily monitoring of their condition, whereas clinicians emphasized the benefits of rapid drug titration and efficient patient management. Key implementation barriers included the resources necessary to support the intervention and lack of physician remuneration.

**Conclusions:**

This study presents results from a real-world implementation assessment of remote titration facilitated by telemonitoring. It is among the first to provide insight into the perception of the remote titration process by clinicians and patients. Our findings indicate that the relative advantages that remote titration presents over standard care strongly appeal to both clinicians and patients. However, to ensure uptake and adherence, it is important to ensure that suitable patients are enrolled and the impact on the physicians’ workload is minimized. The implementation of remote titration is now more critical than ever, as it can help provide access to care for patients during times when physical distancing is required.

**Trial Registration:**

ClinicalTrials.gov NCT04205513; https://clinicaltrials.gov/ct2/show/NCT04205513

**International Registered Report Identifier (IRRID):**

RR2-10.2196/19705

## Introduction

Heart failure (HF) is a common diagnosis affecting at least 26 million people worldwide [1]. It is associated with poor clinical outcomes and high use of health care resources. Large-scale randomized controlled trials (RCTs) have demonstrated that guideline-directed medical therapy (GDMT), optimized to maximal tolerated doses, improves clinical outcomes in patients with HF [2]. However, in clinical practice, large registries confirm that GDMTs are underused, and management of HF tends to fall short when it comes to dose optimization [3,4].

Patient-related factors such as time constraints and financial limitations, physician-related issues such as knowledge of drug therapy optimization, or institution-related logistical issues surrounding clinic visits often complicate the titration process [2,5]. These factors present barriers to timely optimization of vital therapy for patients with HF, which are particularly detrimental, as delays in therapy can lead to significant disease progression that may have been preventable [6].

Telemonitoring is a potential component in the management of HF that allows patients to remotely provide reliable and real-time physiological data for clinical decision support. As such, telemonitoring could be used to facilitate remote titration of HF medications by health care providers. Meta-analyses of telemonitoring studies indicate that telemonitoring has a positive impact on HF outcomes, such as mortality and hospitalizations [7-9], while qualitative assessments of telemonitoring interventions and their acceptance reveal that both patients and clinicians view telemonitoring as efficacious and useful [10-12]. Research on remote titration of HF medications is somewhat limited; however, the results of previous studies indicate that remote titration could be leveraged to garner improvements in GDMT optimization [13-17].

A pilot RCT was conducted at the Peter Munk Cardiac Centre (PMCC), University Health Network (UHN), in Toronto, between January and December 2019. The trial enrolled 42 patients and compared a remote titration intervention, facilitated by telemonitoring data, with standard in-office titration. Within 6 months of enrollment, 86% (18/21) of patients in the intervention group achieved optimal doses versus only 48% (10/21) of patients in the control group. The median time to dose optimization was 7.8 weeks lower in the intervention group, and the number of in-person visits was reduced by 54.5% [18]. The purpose of this paper is to describe a qualitative study that assessed the perceptions and experiences of clinicians and patients with HF participating in the remote titration intervention to identify barriers and facilitators that impact its implementation. The full study protocol and the results of the pilot have been published separately [18,19].

## Methods

### Study Overview

This paper discusses the qualitative component of a mixed methods study aiming to evaluate the effectiveness and implementation of remote titration facilitated by telemonitoring. The study consisted of a pilot RCT and a qualitative study with a purposive sample of participants.

The study was registered on ClinicalTrials.gov (NCT04205513) and received approval from the research ethics boards (REBs) of the University of Toronto (REB number 00036655) and the UHN (REB number 18-5351), where patients were recruited and patient data were stored.

### Study Design

The study was conducted in a Heart Function Clinic (HFC) at PMCC. The RCT compared a remote titration strategy, which used data from a smartphone-based telemonitoring system versus a standard titration program consisting of in-office visits. The qualitative study consisted of semistructured interviews conducted with clinicians and patients allocated to the intervention arm during the RCT.

### Medly Telemonitoring Program

Medly, a telemonitoring program for patients with HF launched at UHN in 2016, was chosen to facilitate remote medication titration in this study. Medly enables patients to take daily clinically relevant physiological measurements with wireless home medical devices in addition to answering symptom questions through a mobile app. The measurements are transmitted to the mobile phone and then to a data server. If there are signs of their status deteriorating, an individualized alert generated through a rule-based algorithm is sent to a clinician at the HFC through an email. Clinicians are also able to view alerts and their patients’ telemonitoring data through a secure web portal. Studies performed to evaluate Medly found improvements in patient health outcomes, as well as high patient and clinician satisfaction [20-22].

### Remote Titration Intervention

Data reported via Medly were used to perform medication changes every 2 weeks through communication between the nurse coordinator and patients over the phone. Details regarding the remote titration process can be found in the papers outlining the study protocol and the results of the pilot RCT [18,19].

### Study Population

A subset of patients randomized into the intervention group and all the clinicians participating in the remote titration program were invited to participate in individual interviews intended to assess their experiences and perceptions of the program on titration completion. Maximum variation sampling [23] was used to select patients representing a range of experiences with the intervention. The patient participants included men and women varying in age, patients who resided at different distances from the clinic, and a patient who chose to withdraw from the intervention.

### Data Collection

Semistructured one-on-one interviews were conducted with patients and clinicians. Interview guides were designed to explore participants’ views on various aspects of the remote titration program. The Chaudoir et al [24] multilevel framework that outlines factors that predict implementation outcomes was broadly used to conceptualize the interview to touch upon the various factors. However, to ensure that generated information was based on the participants’ unique perspectives, questions did not follow specific constructs. Instead, participants were asked open-ended questions to obtain a sense of their comfort with the intervention and its delivery, any concerns or difficulties they may have had regarding the intervention, and whether it met their goals and expectations.

Interviews lasted 20-45 minutes and were conducted in a quiet and private space within the clinic or over the telephone, depending on the preference of the participant. Before the interview, participants were informed that notes will be taken and that the interviews will be audiotaped for data analysis. Interviews were transcribed verbatim.

### Data Analysis

Conventional content analysis [25] was used to analyze the transcribed interviews and coding was performed via the software NVivo version 12 (QSR International). A conventional approach was selected to gain direct information from study participants, without imposing preconceived categories or theoretical perspectives, and to ensure that knowledge generated from the content analysis is based on the participants’ unique perspectives [26].

Specifically, following familiarization with the data, initial codes were generated by 2 researchers (PW and VA) independently, via standard inductive thematic analysis, allowing the categories and codes to flow directly from the collected data [25]. After the initial round of coding, the researchers discussed emerging codes until consensus was reached. The results were reviewed and refined to identify themes reflecting the issues arising from the data set.

Finally, deductive content analysis was used as the final step to frame the analysis. The deductive analysis used existing theory or predetermined categories to guide the content analysis [25]. Specifically, the themes generated through the content analysis were delineated and mapped following the theoretical framework by Chaudoir et al described below [24]. The mapping to the framework was reserved for the last stage to ensure that the full range of themes emerging from the data was captured under the broader constructs.

### Theoretical Framework

Technology acceptance frameworks often heavily focus on the technology itself and its users. However, even though some of them have a sociotechnical lens, they tend to omit other levels of complexities brought in when technology is nested within the complex context of an organization and the broader system itself. As our intervention was embedded within the HFC at PMCC, the intent was to explore the full range of factors that impacted its implementation. Furthermore, while many different frameworks address the implementation process and implementation outcomes, there is considerable heterogeneity in the constructs that are included and the operationalization of constructs with the measures available to assess them. Some frameworks examine the impact of a single type of factor, such as constructs related to the individual provider (eg, Transtheoretical Model [27,28]) or constructs related to the organization (eg, Implementation Effectiveness Model [29]), whereas the more recent frameworks include a set of multilevel factors or constructs at micro-, meso-, and macrolevels [30-34].

The Chaudoir et al framework [24] was ultimately selected to guide this research, as in addition to innovation-, provider-, organization- and structural-level factors, it includes the patient-level factor and its related constructs. Patient-level associated constructs, such as patient health literacy, health-relevant beliefs, motivation, and personality traits, impact patients’ perceptions of and experiences with the innovation. Thus, this framework, which has been successfully used to guide the evaluation of the largest Canadian heart failure and chronic obstructive pulmonary disease telemonitoring program [12], brings a holistic lens to implementation research, especially for complex interventions.

## Results

### Participants

Interviews were conducted with 16 participants (N=16), as outlined in Table 1. Of the 8 (n=8) clinicians invited to participate in the interviews, 63% (5/8 clinicians) agreed to participate and 38% (3/8 clinicians) were unavailable owing to scheduling conflicts. The clinicians (5/8, 63%) consisted of the dedicated program nurse, who participated in the project from the planning stages, 2 cardiologists who were early adopters of the intervention, and 2 cardiologists who were late adopters of the intervention, as outlined in Table 1. The patient interviewees (n=11) included 91% (10/11) of patients who completed the remote titration program and 9% (1/11) who requested to discontinue remote medication titration and exit the study; this 1 patient requested to exit the study, as he was not comfortable with performing medication changes over the phone.

**Table 1:** Overview of clinicians and patients that participated in the semistructured interviews.

Study identifier			Role		Sex		Age (years)		Description
**Clinicians**									
	Clinician 1	Nurse coordinator		Female		N/A^a^		Dedicated program nurse	
	Clinician 2	Cardiologist		Female		N/A^a^		Early adopter (month 1), under 5 years in practice	
	Clinician 3	Cardiologist		Female		N/A^a^		Late adopter (month 3), over 10 years in practice	
	Clinician 4	Cardiologist		Male		N/A^a^		Late adopter (month 4), over 10 years in practice	
	Clinician 5	Cardiologist		Female		N/A^a^		Early adopter (month 1), under 5 years in practice	
**Patients**									
	Patient 1	Patient		Male		59		Greater Toronto Area	
	Patient 2	Patient		Male		62		Greater Toronto Area	
	Patient 3	Patient		Female		60		Remote location	
	Patient 4	Patient		Male		50		Remote location	
	Patient 5	Patient		Male		46		Remote location	
	Patient 6	Patient		Male		57		Greater Toronto Area	
	Patient 7	Patient		Female		37		Greater Toronto Area	
	Patient 8	Patient		Female		49		Greater Toronto Area	
	Patient 9	Patient		Male		54		Greater Toronto Area	
	Patient 10	Patient		Female		55		Remote location	
	Patient 11	Patient		Female		57		Remote location	

^a^N/A: not applicable.

More men (9/16, 55%) than women (7/16, 45%) participated in the interviews. This distribution is in line with the overall population of the RCT. The age range of the patient interviewees was 37 to 62 years, with a higher proportion of patients in their 50s (9/16, 55%). Of 11 patients, 6 (55%) resided in or near the Greater Toronto Area, requiring a commute of 1.5 hours or less to reach the clinic, while the remaining 5 (45%) lived farther away from the clinic requiring a commute of more than 1.5 hours. This sample was representative of the patients attending the UHN HFC. Patients are frequently referred to this particular clinic for a heart transplant or mechanical circulatory support device therapy. Therefore, the clinic treats patients from across the province of Ontario and has a higher-than-average proportion of severely ill patients, including very young patients with HF.

### Findings

#### Overview

Interviews revealed that most participants viewed the program positively and thought that the intervention was successfully implemented. However, some factors that can hinder implementation success were identified as well. Results are summarized in accordance with the 5 levels of the Chaudoir multilevel framework [24]: innovation-, patient-, provider-, organizational-, and structural-level factors.

#### Innovation-Level Factors

A key aspect of the intervention, which differentiated it from standard care, was that it largely relied on communication via technology. Both patients and clinicians were satisfied with the use of the telephone as the mode of communication for medication titration purposes:

[I] have a number for the nurse, and anytime if I want to talk, I can contact her. Like in case we make an increase, and it is not agreeing with me, I can let her know... I can always contact her.

Patient #11

The intervention also relied on data that were reported daily by patients, and clinicians found this suitable owing to the comprehensive monitoring that this approach facilitated. All the clinicians believed that the daily measurements provided a reliable and timely reflection of the patients’ conditions. Some stated that the intervention made it possible to obtain more comprehensive and accurate data about the patient’s well-being than standard care and found that daily data provided the clinicians with a reassurance that their patients were safe:

In fact, you can make an argument that it’s a more reliable way to know what the effects of your changes are, because, for example, traditionally when you make an adjustment in one of their medications, you get a vital sign assessment in the clinic, you may get the patient to check sometime between now and the next time you see them, or you may not, so I actually found that making adjustments through [the remote titration intervention] and having patients assessed on a daily basis, in their own environment, in many ways was reassuring, as opposed to concerning for the safety components.

Clinician #2

The ease of use of the intervention was another topic commented on by both clinicians and patients, encompassing the smooth integration of the intervention into clinical practice and the patient’s daily routine. The clinicians who participated in our intervention expressed satisfaction with the intervention process and indicated that it provided a plan that was easy to follow, integrated well with clinic practices, and was not onerous. Patients also found the app straightforward and convenient:

It was very well organized. I like the term “slick,” so it flows well with your clinic interactions.

Clinician #3

The app is very straightforward, usually it’s very fast. Once you go through it once or twice, it seems quite easy.

Patient #1

The intervention also presented a relative advantage over standard care, which served as a significant facilitator. The intervention provided a way to overcome the limitation of clinic space. Several clinicians touched upon the fact that the clinic had to cope with a large volume of patients, which sometimes imposed a limitation on how frequently patients could be seen:

I know from my experience that when starting a patient on brand new medication for their heart failure and you bring them into the clinic, no matter how good you are, clinic visits are based on space. It may take you three months to get them anywhere, it just takes longer because of the feasibility of bringing them back, etc.

Clinician #2

Perhaps the strongest implementation facilitators from the clinicians’ perspective were highlighted by 3 themes associated with the usefulness of the intervention: the ability to perform more titrations, rapid achievement of target doses, and optimization of clinic resources:

They have to wait around...that time and that process is of no benefit to them, and the outcome is the same as what Medly Titrate does. And not only that, but you’re also potentially not triaging a patient who does require that, so I think...it’s like balancing the resources, which is a finite amount of space and time in the clinic, to see the patients who need to be seen and optimize the patients who can be optimized remotely.

Clinician #5

#### Patient-Level Factors

The benefits that patients derived from the intervention played an important role in the uptake of the intervention, while certain individual patient preferences acted as barriers. Primarily, all the patients indicated that they favored the intervention, as it allowed them to avoid clinic visits. Most patients noted that it was preferable, as it eliminated the expenses associated with visits to the hospital. Importantly, 5 patients indicated that they resided too far away from Toronto and would not have been able to attend visits at the required frequency. They were only able to undergo guideline recommended biweekly titrations through the remote titration program:

I am about 3 hours away from [the HFC] so the drive down takes a long time, and there’s a lot of waiting involved...That was the main reason I wanted to be in the study, so I wouldn’t have to come in.

Patient 4

Conversely, individual preferences, such as a preference for face-to-face contact, or the lack of desire to perform daily measurements over a prolonged period, highlighted potential barriers to implementation. Notably, 1 patient requested to discontinue remote medication titration and transfer to standard care, as he was not comfortable with performing medication changes over the phone:

[I] wait until I see the cardiologist in person and then I start switching the medication. I just don’t go ahead and do what they tell me to...the recommendation is made and then I do my research and then I see the doctor in-person, and we talk about it and then we make the decision.

Patient #9

In fact, all clinicians noted that the success of the intervention depended on the enrollment of suitable patients. The suitability of the patients depended not only on their medical characteristics and their conformity with the inclusion criteria but also on certain personal traits such as the ability to properly understand the information and instructions that they were receiving and act on them:

I think that it’s always about the right patient. So, they have to be a patient who has a degree of understanding and being able to follow directions...you can generally tell which patient won’t.

Clinician #2

#### Provider-Level Factors

Interviews revealed that the workload associated with the intervention served as a potential barrier to implementation. In our study, the nurse coordinator was responsible for the preparation of reports summarizing the patients’ data and condition since the last checkpoint, as well as the implementation of medication changes prescribed by the physicians, and their communication to the patients. This streamlined the physicians’ involvement in the intervention. However, several physicians noted that if the nurse coordinator were absent, the intervention became quite time consuming for them, which would present a significant barrier to implementation:

I think that on the week that [Medly nurse coordinator] was gone there was quite a bit of extra work, so I think it’s super important to have [Medly nurse coordinator], or someone like [Medly nurse coordinator], all the time...it’s very time-consuming for us physicians if we don’t have somebody to take care of it.

Clinician #4

Another provider-level factor impacting implementation was the clinicians’ preparedness to implement the intervention. Of the 5 physicians, 2 (40%) indicated that the information that they received in the beginning to help them decide if they wanted to conduct remote titration facilitated by Medly, was sufficiently comprehensive and motivated them to try the intervention:

I read the email that was sent, circulated in the beginning about to study...everything that I needed was there, to figure out whether I want to participate and enroll patients, and it had what it was doing, so I was committed to enrolling patients that I thought would benefit from it.

Clinician #5

However, 2 physicians pointed to initial uncertainty regarding the way that the intervention would work. This uncertainty impacted their intent to try the intervention in the early stages of the program:

I think my initial concern was trying to figure out how it all works. How am I going to remember what the patients are on? ...and how am I going to be prompted to make any changes? …and the format where I get a prompt from the [nurse] coordinator seems to work well.

Clinician #3

#### Organizational- and Structural-Level Factors

The availability of institutional resources in the form of dedicated nursing staff support served as a substantial implementation facilitator. However, the costs associated with securing such staff, as well as physician remuneration, were perceived as significant barriers for sustaining the intervention beyond the trial period. It is important to note that the physicians participating in this study did not receive any remuneration and performed all the work voluntarily. It was noted that in the long run, the lack of compensation for services performed by clinicians remotely could serve as a deterrent and could impede the extent to which the intervention would be used. The arrangement of remuneration was thought to be necessary to ensure extensive physician buy-in:

I think technology implementation is feasible, I think buy-in from the physicians and the right patients is definitely feasible, and I think that that is the one barrier that could perhaps irk some people about its widespread implementation, which is “how do I get compensation for the work that I’m doing?,” and if that’s addressed, I don’t see why anyone would not think that this is a great thing.

Clinician #2

## Discussion

### Principal Findings

This study aimed to obtain a deeper understanding of the experiences of clinicians and patients with HF taking part in a remote titration program to identify factors that can promote successful implementation of the intervention or hinder it. Most participants expressed favorable views of the intervention. Our multilevel analysis revealed the presence of several facilitators and relatively few barriers. Innovation-, patient- and organizational-level factors predominantly highlighted facilitators, while provider- and structural-level factors shed light on some barriers.

### Innovation-Level Factors

Telephone-administered therapies have recently emerged as an alternative method of treatment delivery. Therefore, it is important to ensure that both patients and clinicians are comfortable with this treatment modality and that it does not have a deleterious effect on the therapeutic alliance. The patients and clinicians participating in our study had a predominantly positive opinion of telephone communication and indicated that their rapport was maintained. This finding is supported by previous studies, which also noted that patients did not find telephone communication discomfiting [35,36]. Active daily monitoring further enhanced the perceived fit of the intervention. Studies have indicated that the success of telemedicine may rely on the capacity of the technology to facilitate prompt detection of clinical deterioration signs, enabling counteractive interventions [37-39]. The findings of our study echo these sentiments. Clinicians believed that the daily measurements provided a reliable and timely reflection of the patients’ condition and allowed for rapid action in the event of any alerts. This management was facilitated by the integration of the intervention within the clinic. The remote titration protocol was implemented by members of the patients’ existing care team. This approach enabled clinicians to rapidly respond to changes in the patients’ condition, potentially contributing to a more effective model of care versus a centralized approach, where a separate team of specialists conducts the telemonitoring and provides treatment recommendations to the patient’s care team [40].

The intervention was found to be easy to use, integrated well into both patients’ and clinicians’ routines, and removed practical barriers to titration. These factors resulted in a favorable user experience for both clinicians and patients and served to facilitate successful implementation. Owing to patient characteristics, such as decreased concentration, memory or vision impairments, and their unfamiliarity with telemonitoring, usability of systems must be clear and simple, as complex systems may cause stress and anxiety [41]. In addition, studies have found that the intensity, complexity, and integration of telemedicine programs into clinical practice are crucial factors that are highly relevant to predetermining the outcomes of interventions [32,42]. All of these factors received predominantly positive feedback in our study.

Perhaps most importantly, the usefulness of the intervention had a crucial impact on its acceptability. Health care professionals and organizations often expect telemonitoring to be one of the solutions for shortages in health care resources, as well as for maintaining or even increasing productivity [41]. Eurlings et al [43] noted that telemonitoring serves 2 important purposes: improving care and reducing costs. These notions were reflected in our findings as well. The ability to perform more titrations and reach target doses faster significantly enhanced the appeal of the intervention to clinicians. On the patients’ end, the intervention eliminated the need to attend clinic visits, which saved them time and money, and in some cases, gave them access to care that they would have otherwise been unable to obtain as frequently.

### Patient-Level Factors

As key stakeholders in all implementation efforts, patients are active agents and consumers of health care from whom buy-in is necessary [24]. The patient-level constructs identified in our analysis highlighted the important role that perceived benefits play in this buy-in. However, these perceived benefits are not universal to all patients, and certain individual preferences, such as a preference for face-to-face contact, highlight potential barriers to implementation.

Other studies have also noted that telehealth should not be viewed as a panacea; there will be groups of patients where telehealth is not in their best interests, and others where its use is unlikely to improve outcomes compared with usual care [38,41,44]. Therefore, alignment between the nature of the program and the patients’ preferences, characteristics, and abilities is paramount [12]. This highlighted a potential change that should be made in our intervention to facilitate more successful implementation. User-related factors are important to note, and it is essential to clearly define the patient population that should be enrolled in the program, beyond clinical inclusion and exclusion criteria. Patient profiles consisting of illness characteristics, comorbidities, cognition, and literacy can serve as a potential helpful tool in matching individual patients with telemonitoring interventions [41]. This will ensure that suitable patients are enrolled in the intervention and could enhance the results of telemonitoring, which in turn will influence the motivation of patients and health care professionals to implement it in daily life.

### Provider-Level Factors

Provider-level factors, namely the intervention’s impact on the clinicians’ workload, highlighted a potential barrier to implementation. Clinicians noted that while the intervention was usually very quick and easy to execute, it became time-consuming in the absence of the nurse coordinator. Owing to the limited time available to physicians, a time-consuming intervention is not likely to be used, and it is important to maintain minimal impact on the physicians’ workload to ensure uptake. This finding is supported by previous research indicating that clinicians expect systems to integrate well into their practice and not impact their workload [38].

The extent of the information provided to physicians at the beginning of the program was another potential factor affecting implementation. In our study, physicians who believed they were prepared to implement the intervention were among the earlier adopters with a larger number of patients enrolled, whereas those who were uncertain of their preparedness came onboard later and subsequently had fewer patients enrolled. This highlighted another potential change that should be made in our intervention to facilitate more successful implementation. The tasks of clinicians involved in the intervention should be clearly delineated and communicated from the start, along with a complete training plan. This would provide a better assessment of the time burden of the intervention and outline its potential impact on the workload of all team members. In addition, this will help establish confidence in the intervention and enable providers to feel more prepared to implement it.

### Organizational- and Structural-Level Factors

Finally, structural- and organizational-level factors included several interrelated findings. Dedicated nursing staff support was a strong organizational facilitator, as it provided a consistent point of contact for the patients and streamlined the physician’s involvement in the intervention. Other studies have also noted that dedicated nursing resources significantly contribute to telehealth work [45]. Furthermore, a recent Cochrane review encompassing 7 RCTs, which included a total of 1684 patients with HF with reduced ejection fraction reported that nurse-led medication titration resulted in a 2-fold increase in the number of participants achieving target doses of β-blockers, a 20% relative risk reduction in all-cause hospitalization, and a 34% relative risk reduction in all-cause mortality [2], highlighting the significant role that nurses can play in such interventions. More extensive integration of nursing staff support could facilitate effective implementation and promote sustainability while potentially reducing operational cost.

However, on a structural level, the financial resources necessary to acquire any required additional nursing staff, as well as arrange physician remuneration, serve as potential implementation barriers. Reimbursement remains one of the most considerable barriers to telemonitoring services, and the need to improve reimbursement models has been noted in many studies as a prerequisite for widespread adoption of such interventions [46]. This challenge is a system issue as reimbursement for clinicians providing various telemedicine services is not sufficiently addressed in the current regulatory framework for health care services. Both in the United States and in Canada, regulatory agencies place constraints on the types of providers that can deliver telehealth services (eg, licensure and credentialing requirements), the allowable originating sites, and the eligible services. However, historically, providers do not receive reimbursement for the specific tools they use to deliver care; rather, providers are paid for the care they deliver. Therefore, it may be beneficial to view telemedicine as a modality for delivering health care. As noted by LeRouge and Garfield [47], the key to telehealth success in the future is to view it as an integral part of health care services and not as a stand-alone project. The establishment of reimbursement models can help promote telemedicine from an experimental modality to a standard health service within health care organizations, and the implementation of telemedicine would enable providers to deliver timelier, patient-centered, high-quality care.

### Study Limitations

The results of this study should be interpreted while taking some limitations into account. The patient population enrolled in this study was recruited from a single specialized HFC that had launched the Medly Program in 2016. On the clinicians’ end, their familiarity with telemonitoring, as well as the existing processes for communication of information obtained through Medly, may have mitigated challenges that could have otherwise been encountered and may have contributed to a more favorable perception of telemonitoring. On the patients’ end, the average age of the participants was notably lower than the general HF patient population. As younger patients may be more comfortable with technology, the younger age of our patient participants may have led to higher preference for telemonitoring and remote care compared with the general HF patient population. However, a previous study conducted with Medly found that its ease of use and the availability of supporting services led to higher and more consistent adherence rates in older patients (70 years or older) [48].

It should also be noted that despite the central role of the nurse coordinator, there was only 1 nurse in this study. This represents a limitation, as this qualitative inquiry included 4 physicians and only 1 nurse. As such, thematic analysis may have highlighted the physicians’ perspective and not revealed important themes from the nurse’s point of view. Similarly, there was only 1 patient in the RCT who requested to discontinue remote medication titration. As there were 10 other interviewed patients who favored remote titration, the emerging themes may have predominantly reflected their experiences and perceptions. Overall, it is important to note that the small number of patients and clinicians involved in this study represents a strong limitation of this qualitative inquiry. Although the gathered data are promising, the single-center nature and limited number of participants, preclude us from drawing definitive conclusions. A study with a much larger number of participants is currently underway, which will allow us to collect more data and provide a more comprehensive assessment of the intervention, its implementation, and its acceptance.

### Conclusions

To realize the potential benefits of remote medication titration, complex challenges of integration in real clinical settings must be faced. This study presents results from the real-world implementation assessment of remote titration facilitated by telemonitoring. It is among the first to provide insights into the perception of the remote titration process by patients and clinicians. Although the intervention was predominantly positively received, our study illuminated both facilitators and barriers, and we proposed several improvements, which can lead to more effective implementation in the future. Our findings indicate that the relative advantages that remote titration presents over standard care, such as rapid GDMT optimization and reduction in clinic visits, strongly appeal to both clinicians and patients. However, to ensure uptake and adherence, it is important to ensure that the characteristics and preferences of enrolled patients align with the program and minimize the impact of the intervention on the physicians’ workload. More extensive reliance on nursing staff support, or perhaps even the incorporation of nurse practitioners who are authorized to interpret diagnostic tests and prescribe medications, could mitigate this issue and facilitate effective implementation, while potentially reducing operational cost.

This qualitative inquiry is particularly timely, as the COVID-19 pandemic has highlighted the need to provide safe care for patients at a distance whenever possible. Remote virtual care can play an important role in maintaining the safety of both patients and clinicians, while ensuring continuity of care. This qualitative assessment of the barriers and facilitators, along with the evaluation of the effectiveness of the remote titration program, represent the first steps in research that can lead to wider implementation and adoption of remote titration in a population that can greatly benefit from it, both under regular circumstances and particularly during challenging times such as the COVID-19 pandemic.

## Abbreviations

GDMT: guideline-directed medical therapy
HF: heart failure
HFC: Heart Function Clinic
PMCC: Peter Munk Cardiac Centre
RCT: randomized controlled trial
REB: research ethics board
UHN: University Health Network
